# Comparison of the three waves of avian influenza A(H7N9) virus circulation since live poultry markets were permanently closed in the main urban areas in Zhejiang Province, July 2014‐June 2017

**DOI:** 10.1111/irv.12532

**Published:** 2018-02-21

**Authors:** Wei Cheng, Xiaoxiao Wang, Ye Shen, Zhao Yu, Shelan Liu, Jian Cai, Enfu Chen

**Affiliations:** ^1^ Zhejiang Provincial Centre for Disease Control and Prevention Hangzhou China; ^2^ Department of Epidemiology and Biostatistics University of Georgia Athens GA USA

**Keywords:** comparison, epidemiological characteristics, H7N9 virus

## Abstract

**Background:**

The sudden increase in the number of human cases infected with avian influenza A(H7N9) virus after September 2016 raised global concern.

**Objectives:**

To assess the changes in epidemiological characteristics of H7N9 cases since the massive closure of live poultry markets (LPMs) in the main urban areas in Zhejiang province.

**Methods:**

We used descriptive statistics to compare epidemiological characteristics of the three distinct waves of H7N9 cases in Zhejiang province. The rural or urban cases were defined according to the location where the patients had exposure within 2 weeks before illness onset.

**Results:**

Between July 2014 and June 2017, 166 H7N9 cases were reported in Zhejiang province, with 45, 34, and 87 cases reported in the third, fourth, and fifth wave, respectively. Across the three waves, most reported cases were from rural areas. A similar percentage of cases in all three waves reported exposure to LPMs, raising poultry at home or around the house, as well as occupational exposure. Compared to the third (80.00%) and fourth wave (70.59%), a significantly larger proportion of cases from the non‐LPMs closure areas were observed in the fifth wave (89.66%) (*P* = .034).

**Conclusion:**

Epidemiological characteristics of human cases infected with avian influenza A(H7N9) virus had generally remained unchanged since the massive closure of LPMs in the main urban area of Zhejiang province. The sudden increase in the number of H7N9 cases in the fifth wave was mainly attributed to the excessive cases reported from areas where LPMs were not permanently closed.

## 1 INTRODUCTION

Since the first outbreak of avian influenza A(H7N9) virus in humans in 2013, five seasonal epidemic waves have been documented in Mainland China, resulting in 1557 confirmed cases and 605 deaths as of July 25, 2017.^1^ Epidemiological data suggested that avian influenza A(H7N9) infections were associated with exposure to live poultry markets (LPMs).^2^, ^3^, ^4^, ^5^, ^6^ To control its impact, temporary or permanent closure of live poultry markets has been implemented by many local governments, leading to a declining trend of laboratory‐confirmed cases in the first four waves.^7^, ^8^, ^9^, ^10^, ^11^ However, an unprecedented outbreak of human infection with avian influenza A(H7N9) virus, identified as the fifth wave, occurred in China from September 2016, and a number of cases reported in the fifth wave exceeded those reported in the previous waves in China.^8^, ^9^, ^10^, ^12^

Zhejiang province, located in southeast China, has the largest proportion of H7N9 case numbers among all the provinces in China with a total of 305 as of June 30, 2017. In response to the high incidence of human infection with avian influenza A(H7N9) virus, Zhejiang government implemented permanent LPMs closures in the main urban areas among all cities since July 2014, prior to the emergence of the third wave of H7N9 outbreak.^9^, ^13^, ^14^ Despite such efforts devoted to a massive closure of LPMs in central urban areas, Zhejiang province was still seriously suffered from the avian influenza A(H7N9) virus during the fifth wave, with a total of 87 cases confirmed as of June 30, 2017.

With its substantial economic impact to the poultry industry,^15^, ^16^, ^17^ massive closure of LPMs across the nation has not been implemented in China. Instead, the closure of LPMs in the central urban areas, which has been mandated in Zhejiang province since 2014, is considered as a more feasible option for the local governments in practice. Therefore, analyzing the changes in epidemiology of avian influenza A(H7N9) virus in Zhejiang province may provide useful information for future control and prevention of H7N9 in China. In this study, we compared the epidemiological characteristics of H7N9 cases among the latest three waves since July 2014. The findings from this study will allow us to address whether there were any significant changes in the epidemiology of avian influenza A(H7N9) virus since LPMs were permanently closed in the main urban areas, particularly with regard to the fifth wave.

## 2 METHODS

All laboratory‐confirmed cases of avian influenza A(H7N9) virus infection in the Zhejiang province were reported to the China Information System for Disease Control and Prevention. A standardized questionnaire was used to collect information on demographics, exposure history, clinical signs and symptoms, date of onset, date of first medical visit, date of hospitalization, date of viral infection confirmation, and date of antiviral treatment. For exposure history, we interviewed the cases regarding their activities: (i) visiting LPMs, (ii) raising poultry at home or around the house, (iii) occupational exposure, and (iv) touching sick or dead poultry within 2 weeks before illness onset. Those who answered yes to at least one of:(i) chronic pulmonary disease, (ii) hypertension, (iii) diabetes, and (iv) cardiovascular disease were considered as having underlying conditions.

### 2.1 Definition of urban and rural cases

In this study, the rural or urban cases were defined according to the location where the patients had exposure within 2 weeks before illness onset. If a case had exposure in both urban and rural areas, we defined its exposure location based on the exposure's risk to onset, following the order of LPMs, occupational exposure, and finally raising poultry at home or around the house. We have chosen a somewhat different definition of case location because we believed that the exposed location can reflect the source of infection more accurately and that location should be where we need to take control measures. However, we also conducted the exposure analyses by defining the rural/urban cases according to the location of residence to make better comparison with most of the previous studies.

### 2.2 Definition of the three waves

Based on the date of onset, the third wave in Zhejiang Province was defined from November 1, 2014, to May 31, 2015, followed by the fourth wave starting from September 1, 2015, and ceasing on June 30, 2016, and the fifth wave which started on September 1, 2016, and continues into the date of this analysis.

### 2.3 Ethical approval

The National Health and Family Planning Commission ruled that the collection of data for laboratory‐confirmed cases of avian influenza A(H7N9) virus infection was part of a continuing public health investigation of an emerging outbreak. The study was therefore exempt from institutional review board assessment.

### 2.4 Statistical analysis

The means and standard deviations, or medians and ranges were obtained for continuous variables, and characteristic percentages were calculated for categorical variables. Nonparametric tests, including Kruskal–Wallis tests, were used to compare continuous variables such as times between illness onset and other dates of interest. Cochran–Armitage trend test, chi‐square, or Fisher's exact tests were used to analyze categorical variables for the three waves. SAS9.2 (SAS Institute, Cary, NC, USA) was used for analyses. The level of significance was set at 0.05.

## 3 RESULTS

### 3.1 Epidemiology

**Figure 1:**
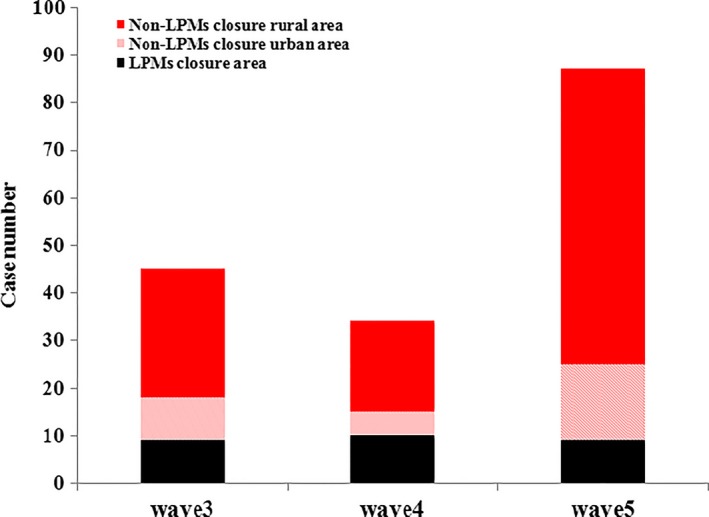
Distribution of laboratory‐confirmed cases of A H7N9 virus infection in closure and nonclosure rural/urban areas of live poultry markets in Zhejiang province, July 2014‐June 2017. LPMs, live poultry markets

As of June 30, 2017, a total of 166 human infections with influenza A(H7N9) virus were identified during the third, fourth, and fifth wave in Zhejiang Province. In each respective wave, 45, 34, and 87 cases were confirmed. Although there was no statistically significant difference seen among the three waves in death rate of human infections with H7N9 cases, the death rate in the fifth wave was much lower (33.33%) than that in the fourth (38.24%) and third wave (53.33%) (Table 1). No statistically significant differences were observed in terms of age and sex across the three waves. Of the 166 cases, 108 (65.06%) were from rural areas. Rural cases occupy a larger proportion than urban cases in each wave (60.00% rural cases in the third wave, 55.88% rural cases in the fourth wave, and 71.26% rural cases in the fifth wave), but did not significantly differ across the three waves (*P* = .198). The occupation and underlying medical disease did not differ statistically significantly among the three waves. Compared to the third (80.00%) and fourth wave (70.59%), a statistically significantly larger proportion of cases from the non‐LPMs closure areas were observed in the fifth wave (89.66%) (*P* = .034) (Table 1). The number of cases from non‐LPMs closure urban area and non‐LPMs closure rural area in the fifth wave increased dramatically from the third and fourth wave (Figure 1).

**Table 1:** Characteristics of laboratory‐confirmed cases of A(H7N9) virus infection in Zhejiang province July 2014‐June 2017

Characteristics	Overall	September 2014‐August 2015 Wave 3 (N = 45) (%)	September 2015‐August 2016 Wav e4 (N = 34) (%)	September 2016‐June 2017 Wave 5 (N = 87) (%)	*P*
Deaths, N	66 (39.76)	24 (53.33)	13 (38.24)	29 (33.33)	.082
Median age (range)	59 (20‐80)	58 (20‐80)	58 (14‐87)	60 (31‐83)	.455
Age‐group (y)					
0‐19	1 (0.60)	0 (0)	1 (2.94)	0 (0)	.300
20‐39	18 (10.84)	6 (13.33)	1 (2.94)	11 (12.64)	
40‐59	66 (39.76)	19 (42.22)	16 (47.06)	31 (35.63)	
60‐	81 (48.80)	20 (44.44)	16 (47.06)	45 (51.72)	
Sex					
Female	64 (38.55)	15 (33.33)	15 (44.12)	34 (39.08)	.615
Male	102 (61.45)	30 (66.67)	19 (55.88)	53 (60.92)	
Area					
Urban	58 (34.94)	18 (40.00)	15 (44.12)	25 (28.74)	.198
Rural	108 (65.06)	27 (60.00)	19 (55.88)	62 (71.26)	
Occupation					
Farmer	82 (49.40)	21 (46.67)	16 (47.06)	45 (51.72)	.913
Retiree	27 (16.27)	9 (20.00)	6 (17.65)	12 (13.79)	
Other	57 (34.34)	15 (33.33)	12 (35.29)	30 (34.48)	
Underline medical conditions					
Yes	114 (68.67)	27 (60.00)	27 (79.41)	60 (68.97)	.183
No	52 (31.33)	18 (40.00)	7 (20.59)	27 (31.03)	
Whether in the LPMs closure areas					
Yes	28 (16.87)	9 (20.00)	10 (29.41)	9 (10.34)	.034
No	138 (83.13)	36 (80.00)	24 (70.59)	78 (89.66)	

LPMs, live poultry markets.

### 3.2 Temporal distribution

**Figure 2:**
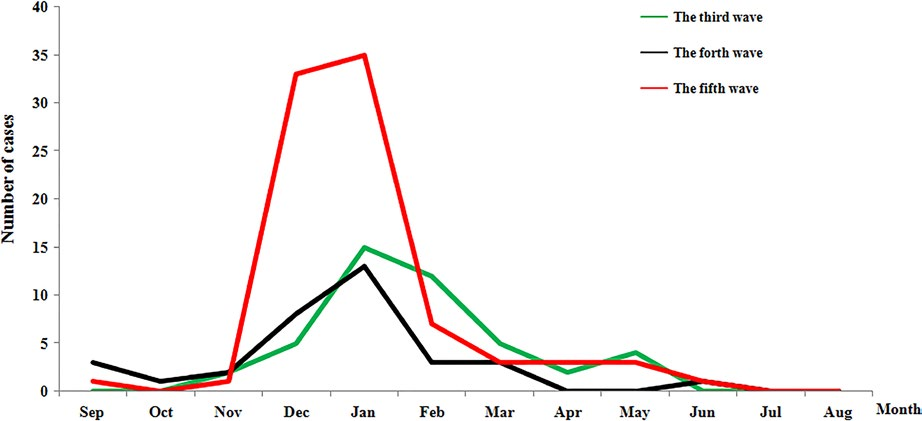
Temporal pattern of laboratory‐confirmed cases of A(H7N9) virus infection in Zhejiang province, July 2014‐June 2017

In both fourth wave and fifth wave, the first confirmed case was reported in September, while in the third wave, the first confirmed case occurred in November. Across all the three waves, the number of H7N9 cases consistently peaked in January, although it was evident that the number of infected cases from the fifth wave was significantly higher than that in the third and fourth wave during the peak period (Figure 2).

### 3.3 Geographic distribution

**Figure 3:**
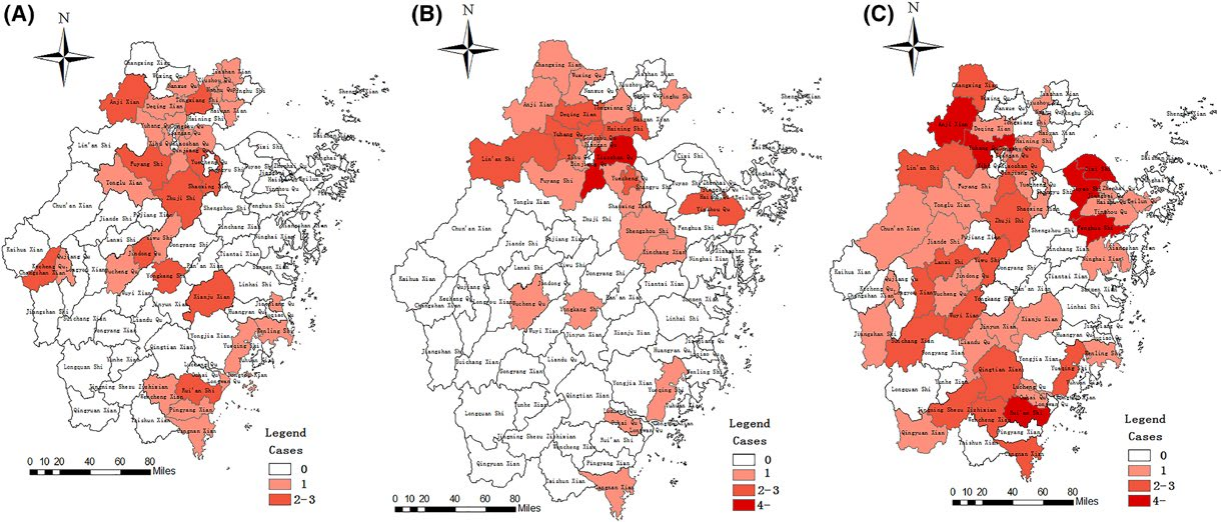
Geographic distributions of laboratory‐confirmed cases of A(H7N9) virus infection in Zhejiang province, July 2014‐June 2017 (A) the third wave, (B) the fourth wave, (C) the fifth wave

The fifth wave has impacted the most cities and districts, with 46 counties in total compared to 32 and 24 counties during the third and fourth wave, respectively. In both third wave and fourth wave, the affected areas were concentrated in the north part of Zhejiang Province. However, cases identified in the fifth wave have spread to a much broader areas of the province (Figure 3).

### 3.4 Exposure pattern

**Figure 4:**
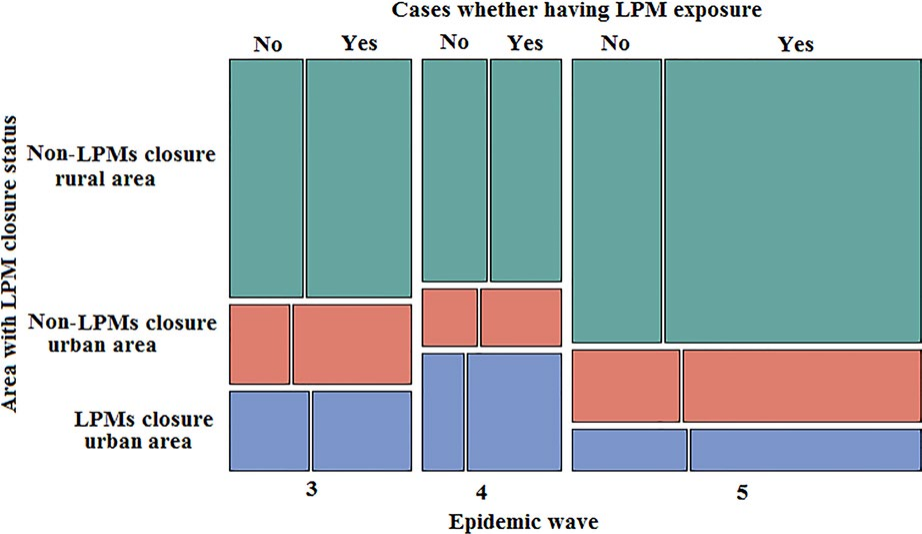
The proportion of H7N9 cases having live poultry markets exposure among areas with different LPMs closure status across the three waves in Zhejiang province, July 2014‐June 2017. LPMs, live poultry markets

A total of 48 cases had multiple exposures, with 46 cases having both LPM and backyard poultry exposure, and the other 2 cases having LPMs, backyard poultry as well as occupational exposures. However, none of them had same exposure history in both urban and rural areas over a 2‐week period. Among both rural and urban cases, there were no significant differences in terms of LPMs exposure, raising poultry at home or around the house, and occupational exposure across the three waves (Table 2). Among rural cases, the exposure to the sick or dead poultry was highest in the fifth wave and an increasing trend was observed (3.70% for the third, 10.53% for the fourth, and 20.97% for the fifth wave). A similar trend was not observed among urban cases (Table 2). Consistently, among cases from LPMs closure urban area, non‐LPMs closure urban area, and non‐LPM closure rural area, over half of them had LPMs exposure across the three waves (Figure 4).

**Table 2:** Exposure pattern of laboratory‐confirmed cases of A(H7N9) virus infection between rural and urban areas in Zhejiang province, July 2014‐June 2017

Exposure pattern	Rural cases				Urban cases			
	Wave 3	Wave 4	Wave 5	*P*	Wave 3	Wave 4	Wave 5	*P*
Visiting live poultry market	16 (59.26)	10 (52.63)	46 (74.19)	.140	11 (61.11)	10 (66.67)	17 (68.00)	.891
Raising poultry at home or around house	18 (66.67)	15 (78.95)	33 (53.23)	.105	7 (38.89)	2 (13.33)	10 (40.00)	.176
Occupation exposure	2 (7.41)	2 (10.53)	6 (9.68)	1.00	0 (0.00)	1 (6.67)	1 (4.00)	.728
Touching sick or dead poultry	1 (3.70)	2 (10.53)	13 (20.97)	.030	2 (11.11)	1 (6.67)	1 (4.00)	.368

When we conducted analyses by defining the rural/urban cases according to the location of residence, the results were consistent to the above analyses except that an increasing trend of exposure to the sick or dead poultry was not observed among rural cases (*P* = .098) (Table 3).

**Table 3:** Exposure pattern of laboratory‐confirmed cases of A(H7N9) virus infection between rural and urban areas (by location of residence) in Zhejiang province, July 2014‐June 2017

Exposure pattern	Rural cases				Urban cases			
	Wave 3	Wave 4	Wave 5	*P*	Wave 3	Wave 4	Wave 5	*P*
Visiting live poultry market	12 (57.14)	12 (54.55)	49 (75.38)	.102	15 (62.50)	8 (66.67)	14 (63.14)	.970
Raising poultry at home or around house	12 (57.14)	15 (68.18)	34 (52.31)	.430	13 (54.17)	2 (16.67)	9 (40.91)	.098
Occupation exposure	1 (4.76)	3 (13.64)	5 (7.69)	.615	1 (4.17)	0 (0.00)	2 (9.09)	.598
Touching sick or dead poultry	1 (4.76)	3 (13.64)	13 (20.00)	.091	2 (8.33)	0 (0.00)	1 (4.55)	.550

### 3.5 Key timeline

**Figure 5:**
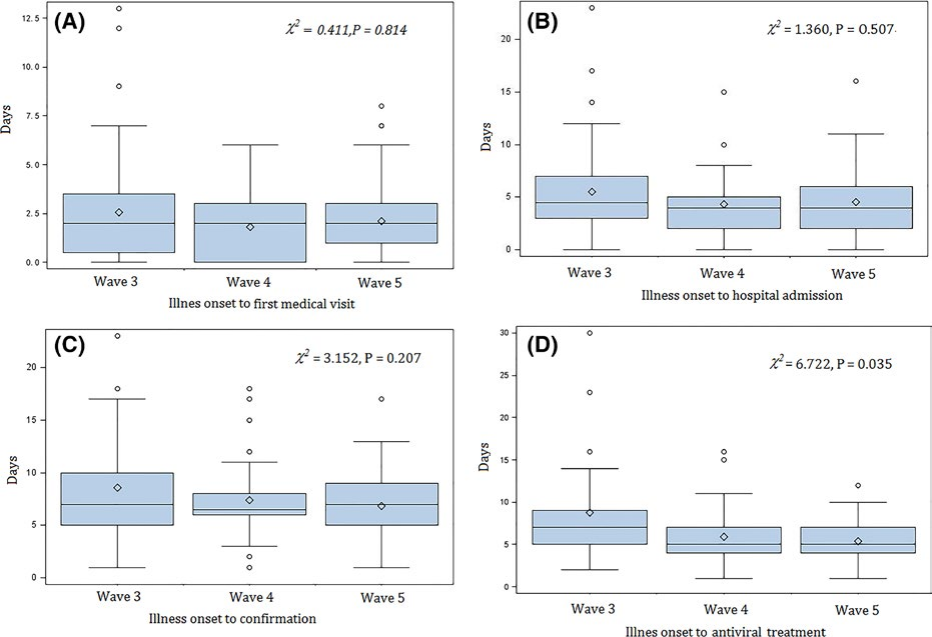
Time to event distributions of influenza A(H7N9) virus infections across the third, fourth, and fifth wave in Zhejiang province, July 2014‐June 2017. (A) Time from illness onset to first medical hospital; (B) time from illness onset to hospital admission; (C) time from illness onset to laboratory confirmation; (D) time from illness onset to antiviral treatment

We found the median time from illness onset to first medical visit (Figure 5, panel A) and the median time from illness onset to laboratory confirmation (Figure 5, panel C) to be consistent across the three waves. The median time from illness onset to hospital admission was 5 days (Range:0‐23 days) for the third wave, 4 days (Range: 0‐15 days) for the fourth wave, and 4 days (Range: 0‐16 days) for the fifth wave with no statistically significant difference (*P* = .507)(Figure 5, panel B). The median time from illness onset to antiviral treatment was shorter for patients detected in the fourth (5 days) and fifth wave (5 days) than that from the third wave (7 days) (*P* = .035) (Figure 5, panel D).

## 4 DISCUSSIONS

Closure of LPMs was conducted to block the transmission of H7N9 and has been considered as the most effective method for restricting the epidemic to date in China.^3^, ^11^, ^17^ Zhejiang Province closed all LPMs in central urban areas concerning the high incidence of H7N9 cases in July 2014, prior to the third wave. Previous studies reported that the number of H7N9 cases in the third wave was significantly lower than that in the first and second wave.^13^ The declining trend was not followed since then, however, with a sharp increase in case number and widespread in geographic distribution during the fifth wave. Despite of the sudden increase in the number of cases in the fifth wave, our comparison of the A(H7N9) virus circulation characteristics among the three waves suggested more similarities than differences. The distributions of age, gender, area (rural/urban), and the exposure patterns remained relatively unchanged, with the exception in the proportion of cases exposed to sick or dead poultry among rural cases being increasing, raising concerns over the control of sick or dead poultry in rural areas. Further, the significantly shortened period between the illness onset and the start of antiviral treatment indicated a better preparation and enhanced awareness of the H7N9 epidemic among health workers in general.

The sudden increase in the number of H7N9 cases during the fifth wave raises global concern.^8^, ^9^, ^12^ The national study showed that the main epidemiological characteristics remained unchanged.^9^, ^10^ Another study conducted in Jiangsu province, the most impacted province in China in the fifth wave, also reached similar conclusions.^18^ Our study also demonstrated that the demographic characteristics and exposure patterns of H7N9 cases had no significant changes during the most recent waves. However, there was a wide geographic distribution of cases as well as new occurring districts/counties in the fifth epidemic comparing with the third and fourth waves, which was also consistent to the above‐mentioned studies.^9^, ^10^, ^18^ Moreover, despite the wide geographic coverage of cases in the fifth wave, the larger proportion of them actually occurred in the non‐LPMs closure areas. This reassured the effectiveness of the LPMs permanent closure approach in the central urban areas. The potential cause for a sudden increase in H7N9 cases in the fifth outbreak was still unclear, but our study indicated that if we had managed to expand the LPMs closure areas, the total number of H7N9 cases in the fifth wave could have been reduced.

Although the government has permanently closed LPMs in the main urban areas within Zhejiang province, new H7N9 cases were still reported from those places. Due to the long‐established Chinese tradition of consuming live poultry, we inferred that illegal live poultry trading in these areas might have existed, as suggested by other studies as well.^16^, ^19^ In addition, massive closures of large LPMs were only implemented in central urban areas, which means many LPMs were still operating in other urban/suburban areas, and residents living in central urban areas could still access those operating LPMs through transportation and be exposed to live poultry, as suggested by our study that over half of cases from non‐LPMs closure urban area had LPMs exposure across the three waves. These findings suggest that in the urban areas where LPMs were required to close, additional efforts devoted to the market management are demanded. Further expansion of the closure areas of LPMs to all the urban areas in the province should be considered if possible.

The closure of LPMs in central urban areas could have resulted in the transportations of contaminated poultry to rural markets, potentially leading to a relative increase in H7N9 cases in rural areas.^13^ However, our study showed that although rural cases still account for majority of the total count, there is not enough evidence to suggest an increasing trend during the latest three waves. Recently, a national study showed that more cases shifted from urban locations to semi‐urban and rural areas.^10^ However,as the definition for rural and urban cases differed in our study, we were not able to make a direct comparison. Nevertheless, previous studies consistently highlighted that strict control measures should be taken in rural areas as well.^10^, ^13^

Our study further revealed that the proportion of rural cases with exposure to dead or sick poultry appeared to be increasing in recent years. The increasing trend of exposure to the sick or dead poultry among rural cases was not observed by defining the rural/urban cases according to the location of residence, though. Compared to urban residents, rural residents usually have higher likelihood of exposure to sick or dead poultry, which were attributed to the popular country lifestyle of raising backyard poultry in China.^5^, ^20^, ^21^, ^22^ The human cases of infection with highly pathogenic avian influenza (HPAI) A(H7N9) virus have been found in Guangxi, Guangdong, and Hunan Provinces during the fifth wave.^23^, ^24^, ^25^ So far, there were no human cases of infection with HPAI A(H7N9) virus confirmed in Zhejiang province. However, a preliminary epidemiological study of HPAI suggested that HPAI A(H7N9) case‐patients were more likely to have had exposure to sick and dead poultry in rural areas.^23^ As rural cases consistently contribute more than half of the new incidents in Zhejiang province, it is essential to raise public awareness of avoiding contacting the sick or dead poultry. To better prepare for future outbreaks, training and health promotion activities aiming at strengthening surveillance in rural areas are necessary.

The time from illness onset to first medical visit, time from illness onset to hospital admission, and time from illness onset to laboratory confirmation did not differ across the third, fourth, and fifth wave, while the time from illness onset to antiviral treatment was significantly shortened after the third wave. The reduced time between illness onset and antiviral treatment was in agreement with an observed declining trend of fatality of H7N9 cases. As the antiviral treatment was recommended as early as possible and considered to be more effective if initiated within 48 hours from illness onset,^26^, ^27^ the median time of 5 days for this period in the most recent wave indicates that there are still rooms for improvements that could potentially reduce future mortality of H7N9 infection by a significant amount. Meanwhile, the strengthening of health education programs and awareness training related to H7N9 should be deemed as essential, as a cost‐effective way of achieving early hospital visits, early diagnostics, and early antiviral treatment.

Our study has several limitations. Firstly, patients’ information was collected through questionnaires and interviews. Hence, subjects’ recall bias, especially on the history of the exposure, cannot be ruled out, which could have introduced bias into the results. Secondly, the limited sample size of H7N9 cases could have restricted our statistical power to detect true differences in epidemiological characteristics across the three waves, especially when subgroup analyses were conducted. Thirdly, A(H7N9) virus infection among the sick or dead poultry was not studied. Therefore, although we found an increasing trend of human cases exposed to sick or dead poultry in rural areas from the analysis, it was not possible to establish a direct association between human cases and H7N9 infections in poultry. Additionally, to better reflect the sources of infection and follow the geographic changes in the H7N9 epidemic more closely, unlike many of the other studies,^5^, ^9^, ^13^, ^20^ we defined the urban or rural cases by subjects’ exposure addresses instead of their current residential addresses. Some of the results may need to be interpreted cautiously, as the exposure addresses were still not necessarily the actual sources of infection, with potential impacts on the calculation of the proportions of the rural and urban cases. Finally, the geography of LPM closure or often blurred distinction between rural and urban areas could have resulted in additional bias that is not fully addressed in the current analyses.

## 5 CONCLUSIONS

Few epidemiological characteristics of the H7N9 had changed since the closure of the LPMs in the main urban areas in Zhejiang province. The sudden increase in the number of H7N9 cases in the fifth wave was mainly attributed to the excessive cases reported from areas where LPMs were not permanently closed. The sizable number of rural cases and the increasing proportion of rural cases exposed to the sick or dead poultry across the three waves suggested the importance of strengthened surveillance in the rural area.

## CONFLICT OF INTEREST

For all authors, none were declared.

## AUTHOR CONTRIBUTIONS

WC XW EC: Conceived and designed the experiments. WC JC: Analyzed the data. SL ZY: Contributed reagents/materials/analysis tools. WC: Wrote the manuscript. YS SL EC: Reviewed the manuscript. All authors read and approved the final manuscript.

## DISCLAIMER

The findings and conclusions in this report are those of the authors and do not necessarily represent the official position of Department of Epidemiology and Biostatistics, University of Georgia.
